# Redefining Bladder Dissection in Robotic Hysterectomy with Previous Cesarean Section: A "Head-On Approach"

**DOI:** 10.7759/cureus.90280

**Published:** 2025-08-17

**Authors:** Rooma Sinha, Bana Rupa, Shilpa Chowdary Peddappolla

**Affiliations:** 1 Minimal Access Surgery Unit, Department of Gynecology, Apollo Health City, Jubilee Hills, Hyderabad, IND; 2 Department of Gynecology and Robotic Surgery, Apollo Health City, Jubilee Hills, Hyderabad, IND

**Keywords:** bladder dissection, da vinci surgical system, head-on approach, previous cesarean section, robotic hysterectomy

## Abstract

Intraoperative adhesions, particularly between the bladder and cesarean scar, as well as between the uterus and anterior abdominal wall, are commonly encountered in patients with a history of cesarean sections. These adhesions substantially elevate the risk of severe intraoperative complications, including bowel or bladder injuries, during hysterectomy. The incidence of bladder complications is significantly lower in patients with one or two prior cesarean sections compared to those with three or more. Moreover, the likelihood of inadvertent cystotomy is several times higher in patients with three or more cesarean sections than in those with no prior cesarean sections. Here, we report a simple, safe, and reproducible *Head-On* approach for bladder dissection. This innovative technique, developed by us, is particularly effective when the lateral window cannot be identified. With the rising incidence of cesarean sections, surgeons are increasingly encountering dense bladder adhesions during hysterectomies. The Head-On technique was developed specifically for bladder dissection in such cases to minimize the risk of urogenital tract injuries.

## Introduction

Cesarean section is the most performed surgery for women globally. In India, the cesarean section rate has risen from 16.72% in 1988-1999 to 21.50% in 2023 [1]. This increase presents surgical challenges, particularly due to the dense bladder adhesions at the cesarean scar encountered during hysterectomy. Dense adhesions can lead to unintended cystotomy, extended operative time, and increased blood loss, all of which contribute to higher morbidity [2]. The da Vinci system's wristed, tremor-free instrument movement offers a distinct advantage for surgeons in addressing this challenge.

Here, we report a simple, safe, and reproducible *Head-On* approach for bladder dissection. The *Head-On* approach for bladder dissection is particularly effective when the lateral window cannot be identified. This technique, which is only feasible with the robotic platform, involves a superior-to-inferior dissection. This technique relies on five key concepts: the three-dimensional (3D) *Head-On* stereoscopic view of the pelvis from the console, the wristed hot shears enabling scissors to work perpendicular to adhesions, tremor filtration providing confidence to use short bursts of monopolar energy near the bladder, human hand-like movement of the fenestrated bipolar to elevate the uterus and stretch bladder adhesions, and the beveled edge of the colpotomizer to define the cervicovaginal junction, identifying the limits of the adherent bladder.

## Technical report

Our approach to surgery

**Figure 1:**
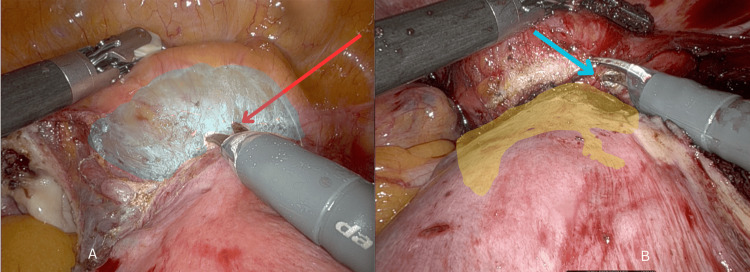
Concept of the Head-On approach. The red arrow indicates the tip of the scissors positioned perpendicular to the line of dissection in the uterovesical fold of the peritoneum, while the blue arrow marks the tip of the scissors perpendicular to the line of dissection in the bladder.

The surgical approach follows the *Sinha-Apollo technique* [3]. Efficient uterine manipulation is achieved using the Rumi uterine manipulator system (Cooper Surgical, Trumbull, CT). The beveled edge of the KOH cup helps identify the cervicovaginal junction. For bladder dissection, the instruments used include fenestrated bipolar forceps (80 watts, Level 3) and hot shears with monopolar current (Coagulation: 120 watts, Level 3; Cutting: 150 watts, Level 3). A 30-degree telescope is used, and a sequential strategy is followed to complete the dissection of all left-side ligaments, along with opening the anterior and posterior left broad ligament. The bladder dissection begins with a *Head-On* view (Figures 1A, 1B).

The midline optic port through the console provides the surgeon with a view of the pelvis as if they are sitting at the head of the operating table, viewing the pelvis with hands on either side. This view is not possible in laparoscopy or open surgery, where the surgeon stands beside the patient. The bladder dissection is carried out in the midline, perpendicular to the adhesion line. Short bursts of monopolar current, combined with the rock-steady tremor filtration property of the da Vinci robot, enable confident fiber-by-fiber dissection. The monopolar current aids in separating fibrous bands from the bladder and reaching the correct plane of the utero-vesical ligament. The glistening plane confirms the correct plane of dissection. The fenestrated bipolar forceps, used like a human hand from the left robotic port, grip the uterus and pull it cranially, stretching the adhesions for easier dissection. The beveled edge of the colpotomiser (KOH cup) identifies the cervicovaginal junction, marking the lower limit of bladder dissection. Once the bladder is safely and adequately dissected, the colpotomy is performed to complete the hysterectomy.

Head-On approach combined with the lateral window

**Figure 2:**
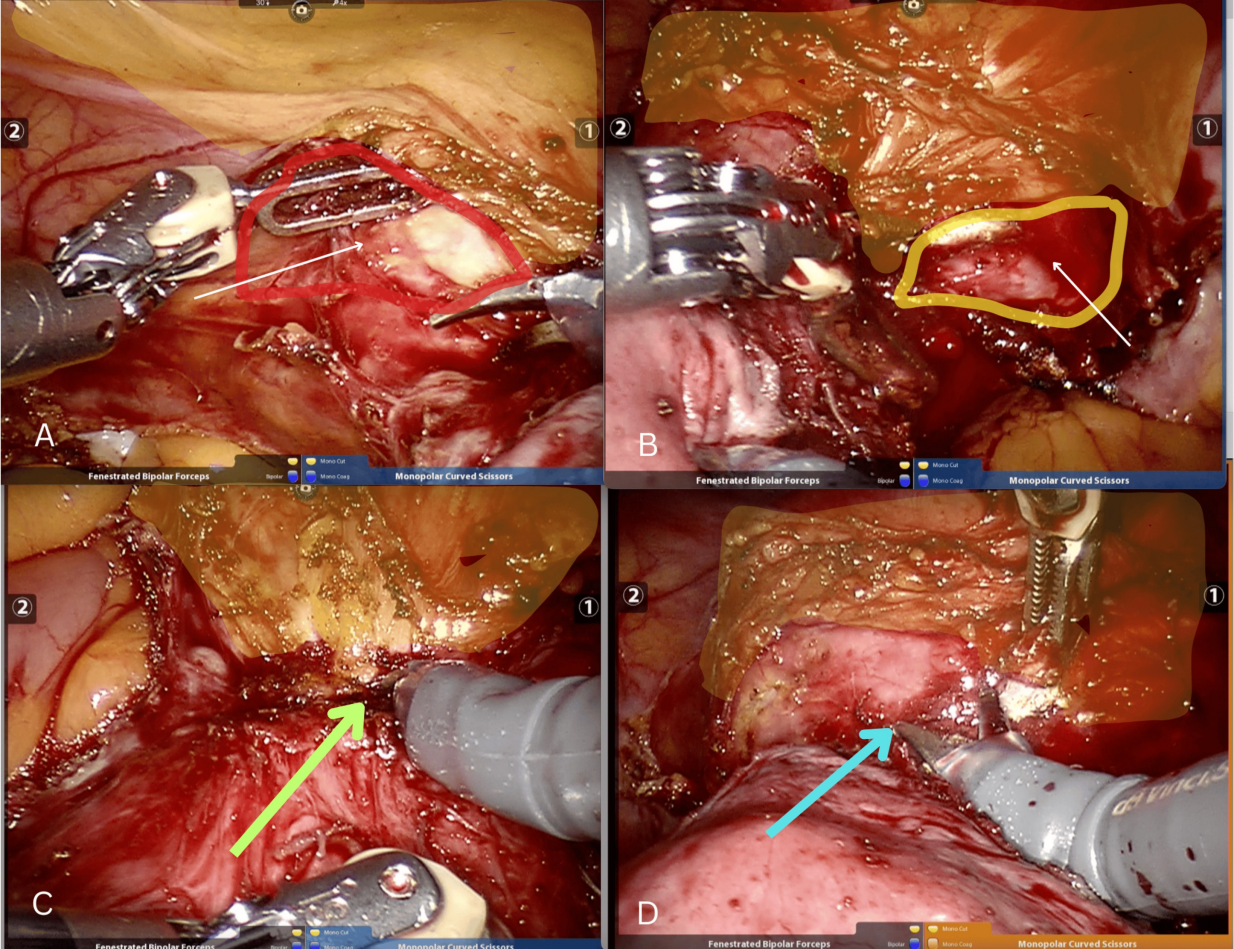
Lateral space with the Head-On approach: (A) left lateral space; (B) right lateral space; (C) dissection of the bladder with the Head-On approach; (D) bladder held with bipolar fenestrated forceps and dissected with scissors using the Head-On approach.

After opening the two leaves of the broad ligament, a lateral space parallel to the bladder pillar, extending down to the cervical region, is identified. This exposes the vesicocervical ligament, confirming the correct plane of dissection. This lateral space has the vesicocervical-vaginal ligament (bladder pillar) and the bladder medially, the broad ligament anteriorly by the cardinal ligament posterolaterally. At this stage, the uterine vessels are exposed, skeletonized, coagulated, and transacted, with the same steps repeated on the contralateral side. Next, the remaining midline bladder adhesions are dissected using the Head-On approach. The fenestrated bipolar is employed to lift and deflect the bladder, while the hot shears are used to transect the fibrous band between the bladder and the previous scar. Typically, this dissection is performed from left to right, unless the fibers are particularly dense, in which case the dissection may begin on the right side. Properly identifying the bladder's upper border is crucial to avoid inadvertent cystotomy. Hot shears can be used either cold or with short bursts of monopolar cutting current. These short bursts help identify the plane between the fibrous tissue and the nascent adipose tissue at the base of the bladder. Dissection becomes easier once the edge of the KOH cup is identified (Figures 2A-2D).

Head-On approach with reverse bladder dissection

**Figure 3:**
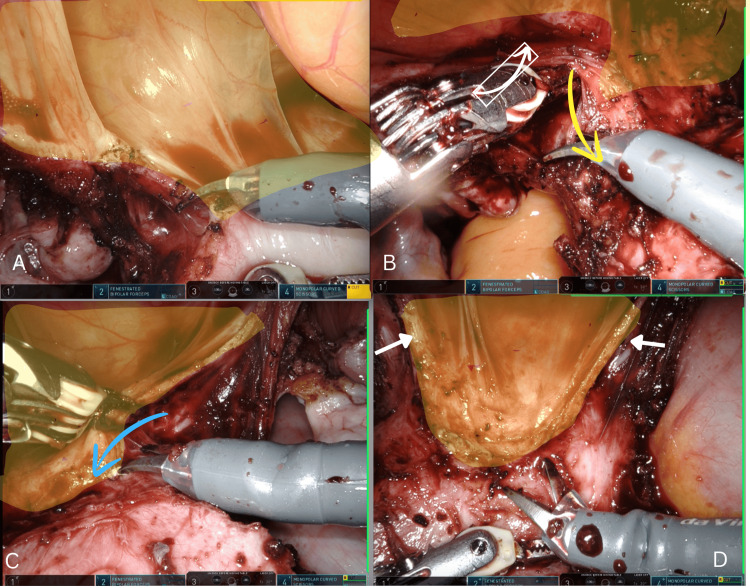
Reverse bladder dissection with the Head-On approach: (A) adherent bladder at the lower segment; (B) space beneath the adherent bladder in the lower segment on the left side; (C) space beneath the adherent bladder in the lower segment on the right side; (D) the Head-On approach of the central adhesion following reverse dissection from both sides.

The second modification technique combines reverse bladder dissection with the Head-On approach. After opening the anterior and posterior broad ligament layers, lateral dissection is carried out inferiorly to reach a nascent plane where the bladder is relatively free of adhesions. This is the same nascent area that would be reached if the space were dissected from the vagina. Once the inferior space is identified, dissection in the vesicovaginal space proceeds from inferior to superior (caudal to cranial) using a reverse dissection approach. The fenestrated forceps are used to lift the bladder, allowing the adhesions to be identified. These bladder adhesions are then dissected and cut using the Head-On approach. This technique facilitates the complete and safe mobilization of the bladder from dense adhesions. Originally described by Nezhat et al. [4] for laparoscopic hysterectomy in patients with previous cesarean sections, we have modified it for use in our robotic hysterectomy cases (Figures 3A-3D).

## Discussion

Bladder dissection in robotic hysterectomy following a previous cesarean section presents a surgical challenge due to dense vesicouterine adhesions and altered anatomy. This can obscure the natural tissue planes and increase the risk of injury to the bladder. In their paper, Nezhat et al. describe a space below the scar that does not have adhesions [4]. This space can be developed and subsequently used in a reverse fashion to dissect the morbidly adherent bladder from the previous scar. The Head-On approach, as described in our study, offers a strategic method for safe and effective dissection in such cases. The robotic platform enhances surgical precision by providing superior magnification, tremor filtration, and improved dexterity. Compared to conventional laparoscopy, robotic assistance with its wristed movements enables a 90-degree approach (Head-On) to the bladder adhesion at the previous scar with distorted anatomy, identifying the tissue planes and minimizing the risk of inadvertent bladder injury. Sheth described a lateral vesicouterine space with minimal adhesions, which can be safely utilized for bladder dissection during vaginal hysterectomy [5].

Several studies have proposed innovative surgical techniques for hysterectomy in cases involving dense vesicouterine adhesions. Yabuki et al. proposed a new classification of the parametrium and its dissection, developing a refined operative approach that has helped reduce blood loss and preserve bladder function [6]. It has been suggested that using the middle rectal artery as a landmark allows for preserving the neural component of the cardinal ligament, thereby maintaining bladder motor function. Additionally, another study outlined a technique to prevent bladder injury during laparoscopically assisted vaginal hysterectomy (LAVH) in patients with vesicocervical adhesions following previous cesarean deliveries [7]. A study on the laparoscopic nerve-sparing radical hysterectomy (LNSRH) technique utilizing the fascial space dissection for cervical cancer found it to be safe, feasible, and relatively simple, with satisfactory recovery of voiding function [8]. Studies have indicated that while the lateral approach was adequate in most cases, it did not allow access to the peritoneal cavity when there were significant adhesions at the vesicouterine peritoneum. In these circumstances, direct dissection of the adhesions on the uterus was necessary [9]. A blunt dissection technique using finger dissection and vessel skeletonization in the posterior vesical wall has been described for managing abnormally invasive placenta previa. This approach was associated with shorter operative time, reduced blood loss, and a lower risk of bladder injury in cases where anatomical landmarks were unclear, particularly in abnormally invasive placentation near the posterior bladder wall during cesarean hysterectomy [10]. Rao and Kade also introduced a six-step laparoscopic technique for dissecting a central uterine band in a ventrofixed uterus, aiming to minimize injury to adjacent structures during repeat cesarean sections and hysterectomy [11]. Consistent with the studies mentioned above, the three approaches we described also focus on safe bladder dissection with minimal inadvertent cystotomy.

By systematically addressing vesicouterine adhesions, surgeons can achieve a safer bladder dissection with fewer conversions to open surgery. Additionally, the technique minimizes excessive traction, which can lead to unintended bladder tears or hemorrhage. However, this approach requires a thorough understanding of pelvic anatomy and proficiency in robotic surgery. In our experience, robotic-assisted bladder dissection in patients with a history of cesarean delivery is feasible and safe when performed by experienced surgeons with advanced training in robotic surgery. However, the learning curve for complex adhesiolysis and bladder mobilization should not be underestimated. Overall, the integration of robotic technology in hysterectomy after prior C-sections enhances surgical precision and patient outcomes. With proper surgical technique, careful preoperative assessment, and intraoperative vigilance, the risk of bladder injury can be significantly reduced, leading to improved recovery and patient satisfaction. Future studies with larger sample sizes and comparative analyses between robotic and other minimally invasive techniques are warranted to further validate the benefits and limitations of this approach.

## Conclusions

With the rising incidence of cesarean sections, surgeons increasingly encounter dense bladder adhesions during hysterectomy procedures. While minimal access techniques are recommended for hysterectomy, genital tract injuries continue to pose a potential risk. Robotic surgery, with its wrist-like, intuitive movements, facilitates bladder dissection within the narrow pelvic space using an approach we term the *Head-On *approach. A thorough understanding of anatomy and the ability to identify the correct dissection planes are essential for safe bladder dissection. The techniques outlined in this study have been developed and refined by the authors, who possess extensive experience in robotic hysterectomies.
